# Cognitive manipulation of emotional and non-emotional information in working memory of patients with depression: a rigid processing style

**DOI:** 10.3389/fpsyg.2023.1183893

**Published:** 2023-06-13

**Authors:** Jiacheng Cui, Jianglin Wen, Dong Wang

**Affiliations:** ^1^Department of Applied Psychology, Binzhou Medical University, Yantai, Shandong Province, China; ^2^Department of Clinical Psychology, Beijing Chaoyang Hospital, Capital Medical University, Beijing, China

**Keywords:** depression, working memory, cognitive manipulation, rumination, cognitive flexibility

## Abstract

**Introduction:**

Cognitive psychology is one of the important perspectives to understand depression. Compared with previous studies, recent researchers increasingly focused on the exploration of the comprehensive cognitive process of patients with depression. The cognitive operation ability of working memory is an important comprehensive cognitive process, which reflects how individuals establish representations. This is the basis for the formation of experience and schema. The purpose of this study is to explore whether there are abnormalities in cognitive manipulation in patients with depression, and to analyze its possible role in the pathogenesis and maintenance of depression.

**Method:**

In this cross-sectional study, depressed patients was enrolled in the clinical psychology department of Beijing Chaoyang Hospital as the case group, while healthy individuals were recruited in the hospital and social meetings as the control group. Hamilton Depression Scale (HAMD)-17, Hamilton Anxiety Scale (HAMA) and rumination thinking scale (RRS) were adopted as measurement tools, and working memory operation tasks were adopted to test each subject, so as to measure their cognitive operation ability.

**Result:**

A total of 78 depressed patients and 81 healthy individuals completed the study. The results showed that the rumination level of the case group was higher than that of the control group, and the difference was significant first; Second, in the “inconsistent” condition, the case group under different stimulus conditions when the response was significantly higher than the control group; Thirdly, the “cognitive operation consumption” value of the case group was significantly higher than that of the control group under the three stimulus conditions, among which, the operational cost value of sadness—neutral stimulus was significantly higher than that of the other two stimulus conditions.

**Conclusion:**

Patients with depression had obvious difficulties in cognitive manipulation of information with different values in working memory, which reflected in the fact that it took them longer time to adjust the relationship between information and established new representations. Among them, patients with depression had a higher degree of cognitive manipulation of sad stimuli, indicating that their abnormal cognitive manipulation had certain emotion specificity. Finally, the difficulty of cognitive operation was closely related to the level of rumination.

## 1. Introduction

Depression is a mental disease that shows an increasing prevalence in modern society, with a low cure rate and a high rate of recurrence. According to the statistics of the World Health Organization (WHO), the incidence of depression ranks first among the four common mental diseases. Studies have shown that by 2020, about 350 million people in the world have suffered from depression (Mendenhall et al., 2017). It is predicted that in 2023, depression will become the second leading cause of death and disability worldwide (Friedrich, 2017). It can be seen that depressive disorder has become a major global public health event, which requires the joint efforts of the whole society to deal with.

Understanding the pathogenesis and maintenance of depression has always been the focus of clinical research on depression, which determines how we can advance clinical treatment of depression. Cognition is considered to be an important perspective for understanding depression. A large number of studies have shown that cognitive factors are one of the important reasons for triggering and maintaining depression (Taylor and John, 2004). Depression as one of the most common mood disorders, its core symptom is a persistent depressive mood, but depression obviously not only changes the patient’s mood, but also changes the patient’s view of the self, the world and the future, that is, the patient’s cognition has changed significantly. Previous studies have shown that cognitive impairment is one of the main manifestations of depression. More than 82% of patients have obvious cognitive dysfunction, and over 40% of patients with depression show two or more kinds of cognitive impairment (Luo and Zhang, 2005). At the same time, some researchers have found that cognitive impairment is a typical residual symptom of depression, and many patients who have reached the standard of clinical remission or even clinical cure still have not had an ideal improvement in their cognitive function, this continues to affect patients’ social functioning and greatly increases the risk of relapse. This suggests that the cognitive deficit in patients with depression is more likely to be a trait factor rather than a state factor, which also reflects the persistence of the cognitive deficit in depression.

As early as the middle of the last century, some researchers have tried to summarize and explain the cognitive characteristics of depression through clinical observation and empirical research, and thus form the cognitive theoretical hypothesis of depression. The classic traditional cognitive theory is the schema theory proposed by Beck in 1967, which emphasized the influence of depressive schema on individuals. The formation of depressive schema is related to the early adverse experiences of individuals, and its themes are generally loss, helplessness and hopelessness (Douglas et al., 2018). After the formation of depressive schema, individuals will have a preference for schema consistency stimuli in the environment, and their sensitivity will be enhanced, which will lead to a large amount of negative information entering the brain of individuals and obtaining cognitive processing (Huang et al., 2021). This theory has important implications for cognitive research in depression, and many researchers have also come to supportive conclusions. For example, in terms of depressive schema, elf-concept coding test (SRET) has been used to confirm that depressive schema is closely related to depression (Kou et al., 2022). In terms of cognitive bias, a large number of research results have also been produced. Taylor found that patients with depression have an attention bias to negative emotional stimuli (Taylor and John, 2004), Joormann used emotional faces as experimental materials and found that patients with depression showed a bias toward sad faces in the dot detection task, which was manifested as a shortened response to the target stimulus after the presentation of negative stimulus (Fritzsche et al., 2010). Mogg proposed in their study that compared with neutral and positive stimuli, depressed patients have a better ability to recognize sad stimuli (Mogg et al., 2006).

In summary, it is not difficult to find that the traditional cognitive theory of depression emphasizes the negative bias in the early stage of cognition. That is, depressed individuals are more likely to capture kinds of negative stimuli in the environment, resulting in a large and rapid influx of such stimuli into the cognitive processing of individuals, and ultimately resulting in individuals being continuously affected by negative thinking.

But with the development of research, more and more studies have come up with inconsistent conclusions. Some researchers suggested that people with depression may not have a negative bias toward early cognition. Some researchers suggested that people with depression may not have a negative bias toward early cognition stage. For example, a meta-study proposed that after improving experimental methods, stimulus presentation and other factors, many researchers used stroop task, dot detection paradigm and other experiments to find that patients with depression did not have an attention bias to negative stimuli compared with normal groups (Peckham et al., 2010). At the same time, some researchers confirmed that there was no directional acceleration of negative stimuli in patients with depression by using the improved dot-probing paradigm and eye movement task (Jenness et al., 2017; Bø et al., 2023). From an adaptive perspective, negative emotional stimuli may indicate threat, especially during social interactions, the cognitive processing ability of sadness, anger and other negative emotional information plays a decisive role in the adaptability of individuals to social life (Veling et al., 2017). Therefore, both depressed individuals and normal individuals may have keen awareness of negative emotional information (LeMoult et al., 2016). All of the above studies suggest that heightened sensitivity to negative information may not be a major cognitive characteristic of depressed individuals.

Recently, more and more researchers have begun to focus on the characteristics of comprehensive cognitive processes in patients with depression, and to explore the role of abnormalities in such cognitive processes in the cognitive mechanism of depression. Among them, the study of working memory is considered crucial. Working memory refers to a memory system with limited capacity that temporarily stores and processes information, which plays a crucial role in individual cognitive activities and is closely related to executive function, problem solving, and other cognitive domains of memory systems (Xue et al., 2022). One of the important functions of working memory is to process stimuli and build representations. In this process, individuals use information in working memory to carry out a series of cognitive operations, so as to form some form of connection between information, or the individual cognitive operation of the original representation of information, adjust the relationship between information to establish a new representation. This cognitive process also lays the foundation for the storage of long-term memory, the formation of individual schemata, and other higher cognitive processes (Weng et al., 2019).

Through the summary of previous studies and clinical observation, we found that patients with depression may not have strong ability to acquire negative information and high sensitivity to negative information (such as high alertness, directional acceleration, ease of processing), but a rigid processing way to negative stimuli, for example, difficulties in disengaging attention from negative stimuli, difficulties in cognitive conversion and difficulties in update (Culpepper et al., 2017; Vanek et al., 2020). These characteristics are closely associated with high rumination thinking in patients with depression (Moretta and Messerotti Benvenuti, 2022). Rumination means that individuals repeatedly think about the causes and possible consequences of negative events, while patients with depression are undoubtedly very easy to fall into negative thoughts, and such repeated negative thinking also causes the cognitive and emotional regulation ability of patients with depression to deteriorate (Farb et al., 2015).

Previous research has pointed to the repetitive, rigid processing characteristics of patients with depression, but the question remains unclear: What causes this rigid processing. What causes depression patients are more likely to fall into the repeated thinking of negative events or negative thoughts, while normal individuals have the ability to timely let themselves out of the negative repetitive thinking through cognitive adjustment, so as to let their emotions return to the normal level. One likely hypothesis is that individuals have inadequate cognitive manipulation of working memory. If the cognitive operation ability of working memory is low, the cognitive operation of information will be obviously difficult, which will make it difficult for the individual to adjust or reconstruct the existing representation in working memory, ultimately make the individual unable to view the problem from a new perspective, and more likely to fall into the repetitive thinking of a certain idea.

In summary, this study hopes to explore the cognitive operation ability of working memory in patients with depression, and mainly tries to answer two questions. First, whether people with depression have significant cognitive operating difficulties relative to healthy people; Second, if depressive patients have significant cognitive operating difficulties, whether such difficulties are emotion-specific. Based on previous studies, we hypothesize that patients with depression have difficulty in cognitive manipulation of both emotional and non-emotional information and that this difficulty is sadness specific.

## 2. Materials and methods

### 2.1. Subjects

The subjects in this experiment were mainly from clinical psychology outpatient department of Beijing Chaoyang Hospital, hospital staff and various communities in Beijing Chaoyang District. From October 2021 to October 2022, 78 patients with depression were randomly recruited from outpatients in the clinical psychology Department of Beijing Chaoyang Hospital by convenient sampling method as the case group. Each patient was recruited by two psychiatrists with more than 10 years of experience in psychiatry according to the Brief International Psychiatric Interview (Mini International Neuropsychiatric Interview) (Sheehan et al., 1998). The screeners asked about depressive mood, mania/hypomania, panic attacks, agoraphobia, social anxiety, obsessive–compulsive symptoms, generalized anxiety, history of traumatic events, psychotic symptoms, anorexia, bulimia, and binge eating. Participants were also asked about their mental health, psychotherapy and medication history. (1) Inclusion criteria of case group: ① Meet the DSM-5 diagnostic criteria for depressive disorder (Schramm et al., 2020), ② the age range is 18–45 years old, ③ right-handed, ④ normal vision or corrected vision, and ⑤ individuals and their families agreed to participate in the experiment. (2) Exclude criteria of case group: ① Schizophrenia, bipolar disorder and other severe mental diseases, ② patients with serious physical diseases, and ③ alcohol dependence, drug dependence patients. At the same time, 81 healthy subjects were randomly recruited as control group by convenient sampling method in hospital and in Chaoyang District of Beijing.

### 2.2. Materials

#### 2.2.1. Hamilton depression scale

Hamilton Depression Scale, developed by Hamilton in 1960, is an other-rating scale used to assess depressive disorder and its severity, and is currently one of the most commonly used scales for clinical depression assessment (Zimmerman et al., 2013). At present, the scale has 17 items, 21 items and 24 items in three versions. In this study, HDMD-17, the most commonly used clinical depression assessment tool, was adopted. This scale has good reliability and validity and is suitable for adult patients with depression. HDMD-17 mainly uses a five-level score, and individual questions use a three-level score. The entire evaluation process generally takes 10–15 min (Zhang J. et al., 2022).

#### 2.2.2. Hamilton anxiety scale

Hamilton Anxiety Scale was developed by Hamilton in 1959 to assess anxiety disorders and their severity (Deligiannidis et al., 2021). At present, it is the most commonly used anxiety disorder other rating scale in psychiatric departments around the world, with good reliability and validity. There are 14 questions in the scale, which mainly includes two parts: somatic anxiety and mental anxiety. The whole assessment process takes about 10–15 min (Zhang Y. et al., 2022). The main role of HAMA in this study is differential diagnosis. Depression and anxiety symptoms are often accompanied, so it is necessary to identify typical patients with anxiety disorders. As HAMA and HDMD overlap in some topics (Mundy et al., 2021), on the basis of referring to the scale evaluation results and clinical consultations, the deputy chief physician of the psychiatry department and clinical psychologists jointly conduct identification. The HAMA score was not analyzed in this study.

#### 2.2.3. Ruminative response scale

Rumination Thinking Scale (RRS) has been widely used to measure individuals’ rumination traits, and the Chinese version of the scale (RRS-CV) has been proved to have good reliability and validity (Han and Yang, 2009; He et al., 2021). The Rumination Scale consisted of 22 questions, each of which described how individuals might react in the face of some life events. Subjects made choices based on their actual situations. The scale adopts four grades, and the final score ranges from 22 to 88 points. The higher the total score, the higher the degree of rumination (Treynor et al., 2003).

#### 2.2.4. WM manipulation task

In this study, a WM manipulation task was used to measure the cognitive manipulation ability of the subjects. We designed three pairs of stimuli: positive—neutral, sad—neutral, and non-emotional—non-emotional. The first two types of stimulus pairs involve emotional stimuli, among which neutral, positive and negative stimuli are taken from the Chinese Face Affective Picture System, which has been verified by numerous experiments and had good reliability and validity in different populations. Among them, we selected 40 positive emotion faces, 40 sad emotion faces, 80 neutral faces, a total of 160 face stimulation images, the number of male and female faces was equal. The selection criteria were: positive and sad faces with recognition >8.5 points and emotional arousal >6 points, while neutral faces with recognition >8.5 points and emotional arousal <3 points. Gender was the same in each pair of face stimuli. In the non-emotional—non-emotional stimulus pairs, we chose four geometric shapes: triangle, square, pentagram, and hexagon. The four geometric shapes were presented in random pairs.

**Figure 1:**
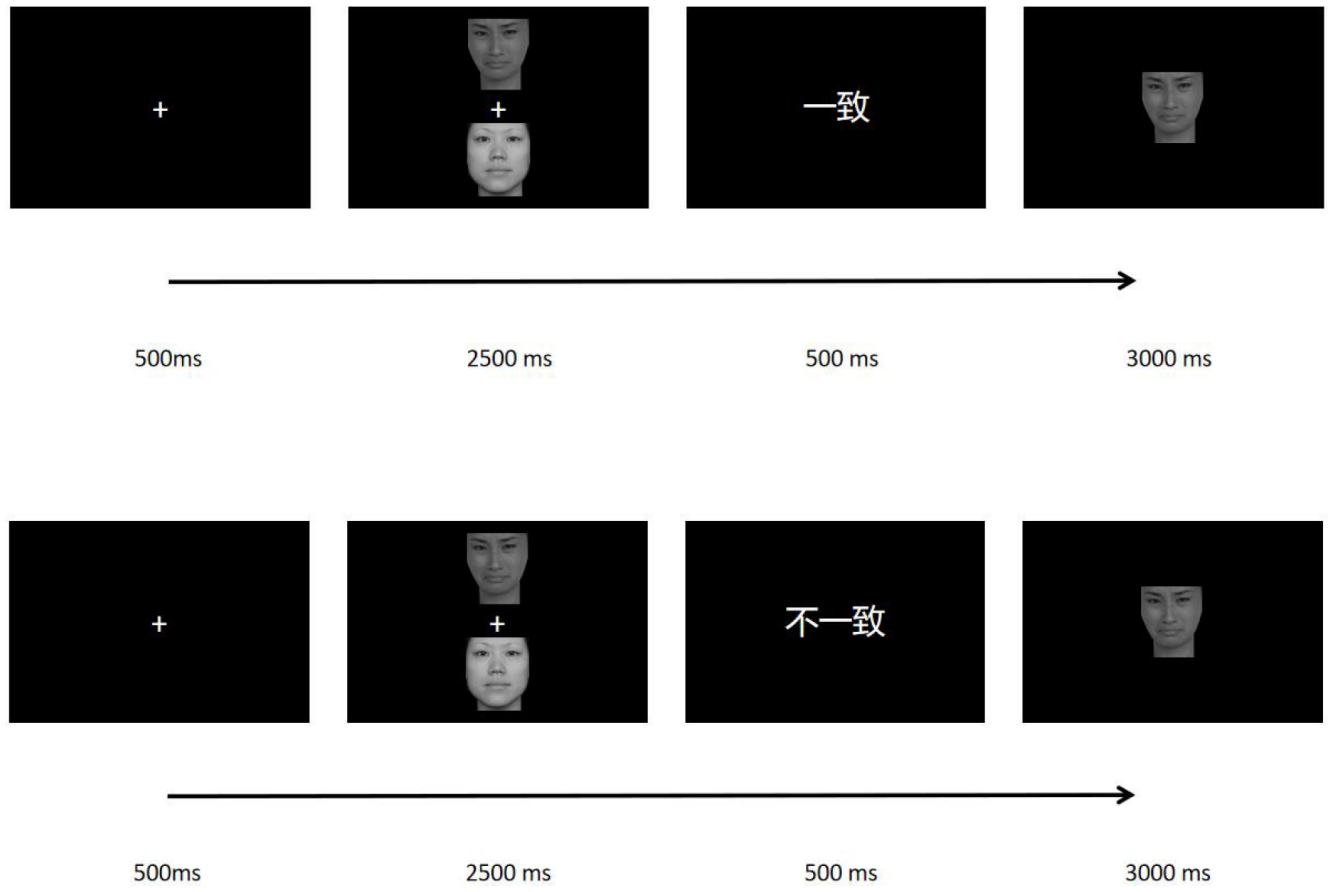
WM manipulation task.

The specific experimental process was as follows: First, a white cross “+” appeared in the center of the screen for 500 ms. Then two stimuli appeared in pairs in the upper and lower halves of the screen. The horizontal position of the central point of the stimulus was 50% of the X-axis, and the vertical position of the central point was 25 and 75% of the Y-axis, respectively. The stimulation in the upper part of the screen corresponds to the A bond, the stimulation in the lower part of the screen corresponded to the B bond. In a pair of stimuli, the location of the two stimuli was random. The stimulus pairs were presented for 2,500 ms, during which the subjects were asked to memorize and master the position of the picture and its corresponding button response relationship. An empty screen of 500 ms was then displayed. A prompt was then presented, which was divided into two conditions, namely “consistent” and “inconsistent.” The “consistent” prompt indicates that the participant still responded according to the original keystroke relationship when the test stimulus appears. The “inconsistent” cue means that when the test stimulus is presented, the participant needed to respond according to the opposite keystroke relationship. After the prompt was presented, follow the empty screen for 500 ms, and finally the test stimulus will be presented. The test stimulus was presented in the center of the screen, and a picture in the previous stimulus pairs was randomly selected. The subjects were asked to make the correct key response according to the previously remembered stimulus position relationship and prompt conditions. The presentation time of the test stimulus was at most 3,000 ms. If the subject makes a key response, the presentation will end; if the subject does not make a response, the next stimulus sequence will be automatically entered after 3,000 ms. The experiment was divided into two blocks, in which the spatial orientation corresponded to the opposite key relationship, so as to balance the influence of spatial position effect on the reaction speed of the subjects. Follow the practice stage in front of each block, so that the subjects were familiar with the experimental process. If the correct rate of the subjects in the experimental stage was not less than 90%, they can enter the formal experiment. The experiment consisted of a total of 360 trails. The specific experimental process was shown in Figure 1.

### 2.3. Data analyze plan

Demographic data of the two groups were collected, includes age, sex, education level, marital status, whether you were an only child and family lived. Questionnaire test and behavioral experiment were conducted by three experienced psychiatrists and clinical psychologists. Each subject strictly followed a standardized procedure. Initially, we recruited a total of 91 depressed patients and 90 healthy individuals as subjects, and only retained the data of subjects with complete scale scores and behavioral results. The final effective data of the case group was 78, the control group was 81. The valid data were input into Microsoft Excel worksheet and then imported into SPSS 22.0 for data analysis. Analysis of variance and independent sample *t* test were used to test the difference. Correlation analysis was also used to investigate the association between cognitive manipulation ability, rumination and depression severity. In a two-sided test, *p* < 0.05 indicates statistical significance.

## 3. Result

There was no significant difference in age, gender, years of education, whether the subjects were the only child, family residence, and marital status between the two groups (*p* > 0.05), as shown in Table 1.

**Table 1:** Comparison of demographic data between case group and control group.

	Case group (*n* = 78)	Control group (*n* = 81)	*χ^2^/F*	*p*
Gender [example (%)]				
Male	35 (44.9)	42 (51.9)	1.673	0.196
Female	43 (55.1)	39 (48.1)		
Marital status [example (%)]				
Married	51 (65.4)	50 (61.7)	0.391	0.530
Unmarried	27 (34.6)	31 (38.3)		
Only child [example (%)]				
Yes	43 (55.1)	37 (45.7)	2.120	0.146
No	35 (44.9)	44 (54.3)		
Place of residence of family [example (%)]				
Urban	60 (76.9)	60 (74.1)	0.152	0.701
Countryside	18 (23.1)	21 (25.9)		
Age (years $\bar{x}\pm s$ )	29.13 ± 8.41	30.24 ± 9.13	0.192	0.988
Years of education (years $\bar{x}\pm s$ )	13.85 ± 3.22	13.23 ± 2.71	0.014	0.839

The reaction of the case group and the control group were analyzed, and the results showed that they were consistent. The reaction time (RT) of the case group and the control group was analyzed. According to the results, the RT of the case group was higher than that of the control group under the condition of consistent prompt. Among them, the response time difference between the two groups under the condition of non-emotional stimulus was significant (*t* = 1.66, *p* < 0.05), while the RT difference of the other two types of stimulus was not significant (p > 0.05). Under the inconsistent prompt condition, the case group had higher RT to non-emotional stimulus, positive—neutral stimulus and sad—neutral stimulus than the control group, and the difference was significant (*p* < 0.01).

In terms of accuracy, there was no significant difference between the case group and the control group under three kinds of stimulus conditions, *p* > 0.05. See Tables 2, 3 for specific data.

**Table 2:** Difference of reaction time between case group and control group.

	Case group	Control group	*t*	*p*
Consistent				
Non-emotion—non-emotion	1458.39 ± 507.86	1430.62 ± 564.41	1.66	<0.05
Positive—neutral	1445.42 ± 488.15	1408.89 ± 504.22	0.98	>0.05
Negative—neutral	1457.76 ± 498.27	1431.90 ± 506.76	0.79	>0.05
Inconsistent				
Non-emotion—non-emotion	1987.72 ± 503.15	1708.31 ± 512.58	6.78	<0.01
Positive—neutral	1952.58 ± 544.08	1695.60 ± 502.51	8.84	<0.01
Negative—neutral	2171.33 ± 501.10	1722.31 ± 504.14	13.48	<0.01

**Table 3:** Difference of accuracy between case group and control group.

	Case group	Control group	*t*	*p*
Consistent				
Non-emotion—non-emotion	89.91 ± 4.68	92.46 ± 5.24	1.02	>0.05
Positive—neutral	89.31 ± 6.72	90.58 ± 7.03	0.89	>0.05
Negative—neutral	90.25 ± 6.11	91.33 ± 5.46	0.67	>0.05
Inconsistent				
Non-emotion—non-emotion	86.01 ± 6.09	90.78 ± 5.58	1.21	>0.05
Positive—neutral	85.24 ± 7.66	88.31 ± 6.79	0.99	>0.05
Negative—neutral	86.71 ± 4.98	89.05 ± 7.20	0.85	>0.05

The core index of this study was “operational cost.” This value reflects that individuals used cognitive resources to form connections between information and establish representations, or the ability to adjust old connections between information to create new representations. In this study, this ability was mainly reflected in the ability of the subjects to adjust the corresponding key response of the stimulus according to the prompt. When we took the response of one type of stimulus under the inconsistent condition and subtract the response of the corresponding type of stimulus under the consistent condition, to get the operational cost value of this type of stimulus. For example: Positive—neutral RT _(inconsistent)_ − Positive—neutral RT _(consistent)_ = operational cost _(positive—neutral)_.

In order to explore the influence of groups and stimulus types on operational cost value, we took groups (case group and control group) as inter-group variables, and presented stimulus types (positive—neutral, sad—neutral, and non-emotional) as intra-group variables for analysis of variance. According to the results, the main effect of the group was significant, *F* (1,157) = 42.89, *p* < 0.01. The main effect of stimulus type was not significant, *p* > 0.05. The interaction between the group and the type of stimulus presented was significant, *F* (2,157) = 10.35, *p* < 0.01.

Further difference analysis showed that the operational cost of the case group was significantly higher than that of the control group *t* = 17.86, *p* < 0.01. The operational cost of the case group was significantly higher than that of the control group under the three presenting stimulus conditions, *p* < 0.01. The operational cost of different types of stimuli presented in the case group was compared in pairs, and the operational cost of the case group under the grief-neutral condition was significantly higher than that under the other two conditions, *p* < 0.01. There was no significant difference between positive—neutral condition and non-emotional stimulus condition, *p* > 0.05. A pairwise comparison of operational cost values of different stimulus strip types in the control group can be obtained. In the control group, there was no significant difference in operational cost value under the three stimulus presenting conditions, p > 0.05. For details, see Table 4.

**Table 4:** Difference analysis of operational cost values of case group and control group.

	Case group	Control group	*t*	*p*
Non-emotion—non-emotion	529.33 ± 113.21	251.11 ± 90.92	10.91	<0.01
Positive—neutral	524.78 ± 102.03	286.71 ± 87.37	13.61	<0.01
Negative—neutral	713.58 ± 124.63	290.43 ± 101.55	29.07	<0.01

In this study, the operational cost of different types of stimuli was analyzed in relation to rumination and depression severity. The results showed that the operational cost of the case group under three kinds of stimuli was significantly positively correlated with rumination thinking level and depression severity, *p* < 0.05. Among them, the operational cost of sadness—neutral stimulus was most closely related to the severity of depression. At the same time, the operational cost in positive—neutral and sad—neutral conditions was significantly positively correlated with the severity of depression. The data is shown in Table 5.

**Table 5:** Analysis of correlation between operational cost, rumination level, and depression severity in case group.

	Rumination	Depression severity
Non-emotion—non-emotion	0.28^**^	0.19
Positive—neutral	0.42^**^	0.35^***^
Negative—neutral	0.18^*^	0.26^**^

^*^p < 0.05, ^**^p < 0.01, ^***^p < 0.001.

## 4. Discussion

The purpose of this study was to investigate the cognitive manipulation of working memory in patients with depression, and to explain the relationship between this cognitive process and depression. As mentioned above, recent research in the field of depression cognition has gradually begun to focus on the relatively late, comprehensive stage of cognition. This trend suggests that the cognitive deficit in depression is a result of the interaction of multiple cognitive domains, rather than merely explained by the abnormality of some basic cognitive functions.

Working memory is undoubtedly a key part of an individual’s cognitive system. Working memory is a memory system proposed by Baddeley and Hitch in 1974. It consists of a central executive system that performs, regulates, and allocates cognitive resources, in addition, it also includes three subsystems, namely speech loop, scene buffer and visual spatial template. These three subsystems are capable of cognitive operation and processing of visual, speech, scene and other information, respectively (Chen et al., 2023). Working memory plays a crucial role in individual cognitive activities, and is closely related to executive function, problem solving, and other cognitive domains of memory systems (Scaini et al., 2021). One of the important functions of working memory is to process stimuli and establish representations. This is a process in which an individual takes information currently in working memory and, through a series of cognitive operations, forms some form of association between the information, or the individual cognitive operation of the original representation of information, adjust the relationship between information to establish a new representation. This important cognitive process also lays the foundation for the storage of long-term memory, the formation of individual schemata and other advanced cognitive processes (Guan et al., 2020).

Previous studies have shown that depressed patients have a high level of rumination thinking and tend to carry out repetitive cognitive processing of negative information in working memory, while depressed mood will constantly provide cognitive materials for such negative thinking (Baune et al., 2014; Miao et al., 2019). These findings point to the repetitive, rigid cognitive characteristics of people with depression. What is not clear, however, is what causes this rigid cognitive trait. Above, we hypothesize that deficits in the cognitive operation of working memory in depressed patients may be the cognitive mechanism behind this cognitive trait, and depressed people may have greater difficulty in cognitive manipulation of sad stimuli. This is likely to be the main reason for the long-term repetition of negative thoughts in depressed patients.

Through the results of this study, we found that, first, compared with the control group, patients with depression have difficulty in cognitive operation of both non-emotional and emotional stimuli, which is a manifestation of universal cognitive operation difficulty. These results indicate that no matter what kind of value stimulation, patients with depression spend more time in cognitive operation, which may be related to tardy and poor cognitive flexibility in patients with depression. A number of previous studies have found that compared with normal groups, patients with depression have delayed reaction speed in visual, auditory and other reaction channels (Baune et al., 2013). Depression will lead to the impairment of various cognitive functions, such as vision, auditory perception, spatial perception, learning association, imagination, hand-eye coordination, thus reducing the cognitive reactivity of the subjects and affecting the information processing speed of the patients (Baune et al., 2013; LeMoult et al., 2015). Therefore, the conclusion of this study is also consistent with previous studies, and we found that there was no significant difference in the overall response between depressed patients and the normal group when presented with consistent conditions, while the difference was significant when the condition was inconsistent, indicating that the higher the task difficulty, the more obvious the difference between depressed patients and the normal group in cognitive operation ability may be. This is also consistent with previous studies. Some researchers have proposed that in comprehensive cognitive tasks, as the difficulty of the task increases, the performance of depressed patients will get worse and worse, indicating that depressed patients are less able to use different basic cognitive abilities to solve problems (Baune et al., 2013; Derntl and Habel, 2017).

Second, in the analysis of the main indicator “operational cost “value, we found that depressed patients showed difficulty in cognitive operation for different value stimuli, this difficulty was reflected in this study as, when receiving “inconsistent” requirements, the subjects needed to spend more time adjusting the previously memorized keystroke response relationship, which led to an increase in the operational cost value. Under the three stimuli, the operational cost value of depressed patients was significantly higher than that of the control group. Through further analysis, we found that in the case group, the operation cost value under sadness-neutral stimulus was significantly higher than that under the other two stimulus conditions, while the operation cost value between positive—neutral and non-emotional—non-emotional was not significantly different. These results indicate that the cognitive operation difficulties of depressed patients have certain emotion-specific characteristics, and the processing of sad stimuli may consume more time of depressed patients. Sad emotional stimuli are most closely related to depressive mood (Derntl and Habel, 2017), and the main themes of depressive mood include powerlessness, hopelessness, self-guilt, which will bring extreme sadness to individuals (Liu et al., 2020). However, it is precisely for this kind of stimuli that depressed patients have greater difficulty in cognitive operation. This also shows that depressive patients do not have high flexibility to sadness stimulation processing, but more rigid and inflexible characteristics.

Thirdly, we found that the cognitive manipulation difficulty in depressed patients was correlated with rumination and depression severity, among which the cognitive operation difficulty stimulated by sadness was most closely correlated with rumination and depression severity. As mentioned above, repetitive thinking of negative thoughts is a key cognitive characteristic of people with depression. From the perspective of adaptive psychology, during the long-term evolution of human beings, they have developed the ability of cognitive regulation, especially the ability to regulate emotions, which enables individuals to recover from the influence of environmental stress in time; otherwise, it will cause adverse effects on their physical and mental health (Barrick and Dillon, 2018). At present, cognitive strategies such as “cognitive reappraisal” and “attention transfer” also reflect the ability of cognitive regulation (Ruohonen et al., 2020). However, people with depression apparently lack this ability. Some researchers have summarized the theme of repetitive thinking in patients with depression through investigative studies, mostly self-guilt, remorse, meaninglessness, pessimism and other related content. In this process of repetitive thinking, patients will gradually feel more sad and even have suicidal thoughts (Kangas et al., 2022). Our results, to some extent, confirm the previous hypothesis that insufficient cognitive manipulation of working memory is a possible cause of the cognitive mechanism of repetitive negative thinking in depressed patients, and that this deficiency is closely related to the severity of depression. Depressed individuals will spend more energy, time and cognitive resources during the cognitive operation of sad stimulus. In the long run, it may cause sad stimulus to be fixed in a rigid representation form and enter into long-term memory and be included into individual experience, resulting in the continuous accumulation and expansion of depressive schema. This may be one of the main reasons why people with depression dwell on negative thoughts, have difficulty regulating their emotions and seeing things from a new perspective.

Finally, we hope to try to explain what role this cognitive deficit plays in the onset and maintenance of depression. Some researchers put forward the “attentional narrowness theory” to explain the principle of rumination thinking, that is, narrow attention scope will make individuals more likely to fall into negative thinking (Yuan et al., 2010), which provides ideas for our interpretation of the cognitive model of depression. Combining the results of this study with those of previous studies, we hypothesize that individuals with narrow attention scope may be more prone to depression, possibly because attention spans affect cognitive span (including cognitive manipulation). When stressors appear in the environment, negative stimuli enter the attention range of individuals. However, due to the limited attention range of individuals, negative stimuli occupy most of the attention space, leading to the neglect of other stimuli. Therefore, when negative stimuli are in the cognitive process, they will naturally be allocated more cognitive resources, leading to the excessive processing of them. This may cause individuals to perform cognitive operations on negative stimuli in a stereotyped response. It leads to insufficient cognitive operation ability in patients with depression, and eventually leads to decreased cognitive flexibility and poor emotional regulation ability in patients with depression, thus causing and maintaining depressive symptoms.

Though we did our best to perfect the study, but there are still limitations in this study. We did not confirm the results of this study with further experiments. An important issue is: Whether the WM manipulation task accurately measures the cognitive manipulation ability of the subjects, whether the difficulty of the task itself and whether the coincidence will affect the accuracy of the results. Other studies that have examined the effect of WM load and task difficulty in depression have not reported that increasing load or difficulty results in a valence-specific deficit in depressed subjects (Levens and Gotlib, 2010). Therefore, we can assume that the results of this study are accurate enough on the whole. We also hope to avoid this limitation with a more rigorous approach in the future.

## 5. Conclusion

We found that compared with the normal group, the patients with depression had obvious difficulty in cognitive operation of working memory, and this difficulty had certain emotion specificity, and the degree of difficulty in cognitive operation of sad stimulus was higher. At the same time, the cognitive manipulation difficulties of the patients with depression were significantly correlated with the level of rumination and the severity of depression. Abnormal cognitive manipulation may play an important role in the pathogenesis and maintenance of depression.

## Data availability statement

The original contributions presented in the study are included in the article/supplementary material, further inquiries can be directed to the corresponding author.

## Ethics statement

The studies involving human participants were reviewed and approved by Ethics Committee of Beijing Chaoyang Hospital. The patients/participants provided their written informed consent to participate in this study.

## Author contributions

JC was responsible for experimental design, testing, data collection, data analysis, and paper writing. JW assisted in experimental design, testing, data collection, data analysis, and paper writing. DW was responsible for paper review. All authors contributed to the article and approved the submitted version.

## Conflict of interest

The authors declare that the research was conducted in the absence of any commercial or financial relationships that could be construed as a potential conflict of interest.

## Publisher’s note

All claims expressed in this article are solely those of the authors and do not necessarily represent those of their affiliated organizations, or those of the publisher, the editors and the reviewers. Any product that may be evaluated in this article, or claim that may be made by its manufacturer, is not guaranteed or endorsed by the publisher.
